# The role of cytosine modification symmetry in mammalian epigenome regulation

**DOI:** 10.1039/d5sc09022a

**Published:** 2025-12-29

**Authors:** Zeyneb Vildan Cakil, Lena Engelhard, Daniel Summerer

**Affiliations:** a Faculty of Chemistry and Chemical Biology, TU Dortmund University Otto-Hahn-Str. 4a 44227 Dortmund Germany daniel.summerer@tu-dortmund.de

## Abstract

5-Methylcytosine (mC) is a key regulatory element of mammalian genomes, and plays important roles in development and disease. mC is predominantly written onto CpG dyads by DNA methyltransferases, and can be further oxidized by ten-eleven translocation dioxygenases (TETs) to 5-hydroxymethyl-, 5-formyl-, and 5-carboxylcytosine. This process results in different symmetric and asymmetric combinations of cytosine forms across the two strands of CpGs, each of which represents a unique physicochemical signature in the major groove of DNA. A comprehensive understanding of the individual functions of oxidized mC modifications can therefore only be achieved by considering both strands of CpG dyads. Here, we provide a brief overview of the current state of knowledge on the sequencing and mapping of individual CpG dyad states, their influence on the intrinsic properties of DNA, and their interactions with chromatin proteins.

## Abstract 2

Cytosine 5-modifications are central to human chromatin regulation. They occur in different combinations in the two DNA strands of CpG dyads, but the functions of individual combinations are poorly understood. We review recent technical developments and biological insights in this area.

## Graphical abstract

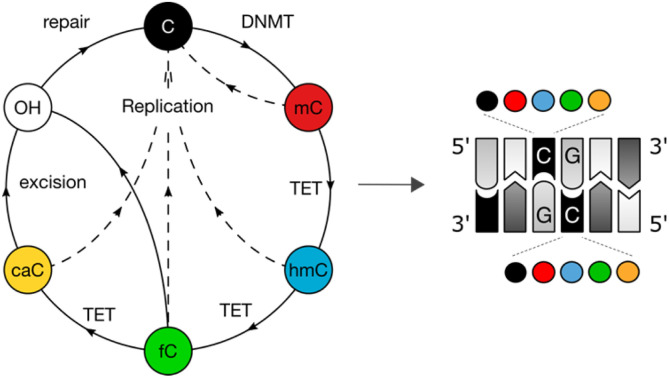

## 1 Introduction

### 1.1 TET-generated cytosine modifications and their existence as symmetric and asymmetric CpG dyad marks

**Fig. 1:**
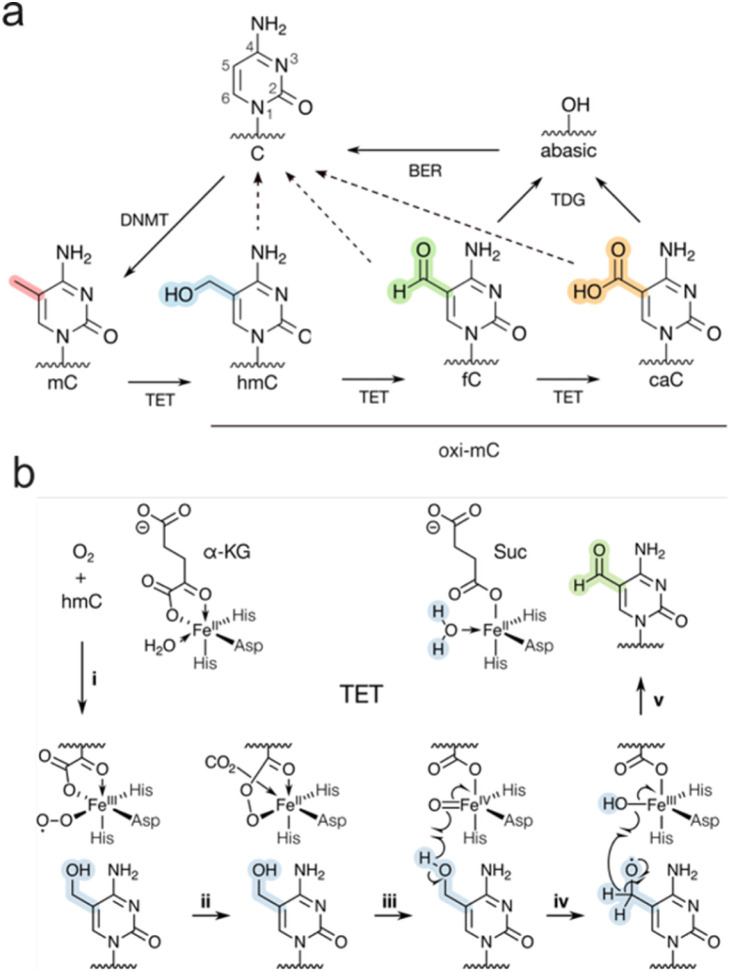
(a) Cytosine methylation and active demethylation in mammals. DNMT: DNA methyltransferase, TET: ten-eleven translocation dioxygenase, TDG: thymine DNA glycosylase, BER: base excision repair. Dotted arrows: passive dilution.^14^ (b) Proposed mechanism of TET-catalyzed oxidation (example shown for oxidation of hmC to fC). α-KG: α-ketoglutarate, Suc: succinate.^18,19^

5-Methylcytosine (mC, Fig. 1a) is the central epigenetic mark of mammalian DNA and acts as a key regulator of transcription, with important roles in developmental and (patho)physiological processes, including genomic imprinting, X-chromosome-inactivation, and cancer development.^1^ mC is predominantly written into CpG dyads by *S*-adenosylmethionine (SAM)-dependent DNA methyltransferases (DNMT),^2^ and is typically associated with transcriptional silencing.^3,4^ mC can thereby be written *de novo* by DNMTa/3b, but can also be maintained over cell divisions by the maintenance enzyme DNMT1 that selectively methylates hemi-methylated CpG dyads generated during replication (Fig. 1a). This makes mC a rather stable, inheritable nucleobase. However, mC can also be passively reversed to C by DNA replication and an absence of maintenance methylation (passive dilution, PD).^3^ Moreover, α-ketoglutarate- and Fe(ii)-dependent ten-eleven-translocation dioxygenases (TETs) can trigger an active demethylation pathway by oxidizing mC to the “oxi-mCs” 5-hydroxymethylcytosine (hmC),^5,6^ 5-formylcytosine (fC), and 5-carboxylcytosine (caC)^7–9^ (Fig. 1b). Base excision repair (BER) can restore C *via* the excision of fC and caC by thymine DNA glycosylase (TDG), and repair of the generated abasic site (active modification – active removal).^7,10^ In addition to this pathway, hemi-modified CpGs containing oxi-mCs seem to compromise DNMT1-catalyzed maintenance methylation compared to hemi-methylated CpG. Consequently, oxi-mC can promote replication-dependent passive dilution of mC *via* active modification and passive dilution (with effects increasing with oxidation state^11–15^). Notably, for reviews discussing these pathways, see ref. 16 and 17.

**Fig. 2:**
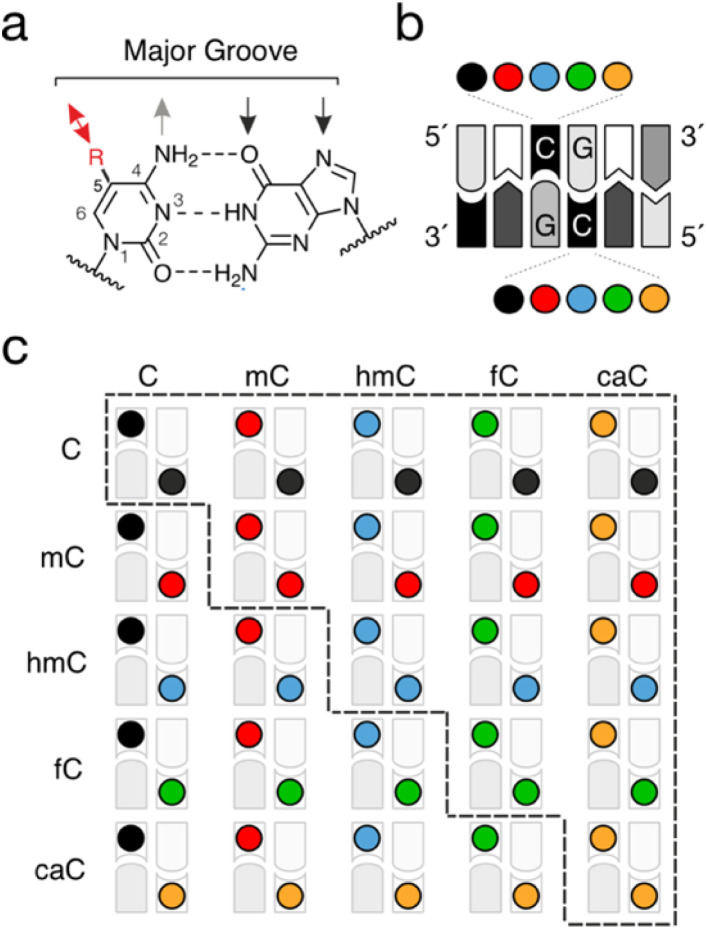
Combinations of cytosine modifications in the two strands of CpG dyads. (a) Chemical information displayed in the major groove of dsDNA. Black and grey arrows denote hydrogen bond acceptors and donors, respectively. Red arrow shows alternative interactions of cytosine 5-substituents. (b) Schematic view of a CpG dyad with possible combinations of cytosine nucleobases. (c) All theoretically possible combinations from (b). Note that 15 different combinations occur within the context of the CpG itself (marked by a dashed line), whereas 25 combinations can occur overall when considering the CpG's sequence context. Color code as in Fig. 1a.

Cytosine modifications can occur in a strand-symmetric and -asymmetric fashion in the double-stranded genome. Whereas non-CpG (CpH) methylation (and partially hydroxymethylation) can occur at certain levels in a tissue-specific manner and is inherently asymmetric,^20,21^ the palindromic nature of the CpG dyad itself allows many different combinations of cytosine modifications to be presented in the DNA major groove, and thus provides rich symmetry information. Indeed, whereas the aforementioned maintenance methylation of hemi-methylated dyads (“mC/C”, for the top and bottom strands, respectively) represents an evolved mechanism to maintain them in a symmetric mC/mC state, the oxidation of mC to hmC, fC and caC by TETs occurs in a step-wise and non-processive manner,^22–25^ and theoretically can give rise to fifteen (mostly asymmetric) modification combinations (Fig. 2a). Whereas TETs exhibit substrate preferences in view of pre-existing CpG modifications in dsDNA,^24^ they can also act on ssDNA, a process that would be inherently independent of modifications of the opposite strand.^26^ Whereas part of these dyads may not occur in appreciable numbers in genomes owing to the low levels of fC and particularly of caC (see below), this nevertheless creates a complex landscape of combinatorial CpG marks across the genome. In particular, dyads involving the most frequent cytosine nucleobases C, mC and hmC are expected to occur at significant levels in stem cells and neurons,^16^ and unique genomic distributions have been observed in first pilot studies (see below).^27–29^ Each individual dyad thereby presents a unique combination of 5-substituents in the major groove, and may uniquely affect the physicochemical properties of dsDNA, as well as interactions with nuclear proteins. The dyads thus represent unique signals with distinct regulatory effects that may arise from dedicated, dyad-specific reader protein interactions, or simply from a unique modulation of pathways relying on a selective recognition of symmetric C/C or mC/mC dyads.

A comprehensive understanding of the individual CpG dyad's functions requires a broad knowledge of their properties as a basis, including their direct effects on the structure and physico-chemical properties of DNA, their genomic levels and locations (including tissue dependence and dynamics), and their individual interaction profiles with the nuclear proteome. We here review the most recent developments in these fields that have helped to shed light on this question and that will guide future studies.

## 2 Simultaneous sequencing of C, mC, and hmC for mapping symmetric and asymmetric CpG dyad states in mammalian genomes

Understanding individual CpG dyad state functions depends on the ability to map their genomic locations. The overall genomic levels of oxi-mCs differ significantly between each other, are tissue-dependent, and can be highly dynamic (reviewed in ref. 30). For example, whereas mC is distributed rather evenly among somatic cells, oxi-mC levels are particularly high in embryonic stem cells (ESC) and neurons (but low in other somatic and particularly many cancer cells), with hmC showing the highest levels in neurons (>10–20% of the levels of mC^30^). In addition, hmC can also show high stability.^31^ In comparison, fC and caC show far lower levels (∼2 and another 1–2 orders of magnitude lower than hmC in mouse ESC (mESC), respectively^30^). These general differences will also translate into different levels of the individual CpG dyad states, which should be considered when judging the physiological relevance of a particular state. A large number of methods have been introduced for sequencing and mapping of oxi-mCs in general (reviewed in ref. 32 and 33), and have enabled detailed maps and correlations with particular regulatory regions. Briefly, hmC is typically found enriched in active enhancers and gene bodies, and in the latter correlates with active transcription (fC and caC are also enriched in gene bodies of actively transcribed genes). In contrast, low levels of all oxi-mCs are typically found in regions surrounding transcriptional start sites of active promoters.^34–38^

However, most aforementioned methods provide selectivity only for single or grouped, but not for each individual cytosine form in a given sequencing run. This limits their analytical value to statistic assumptions rather than accurate determinations of the actual CpG dyad states. Nevertheless, such maps generally indicated that hmC is typically asymmetric.^37–40^ Moreover, an early single molecule imaging study identified hmC/mC as a frequent modification in mouse cerebellum DNA.^41^

**Fig. 3:**
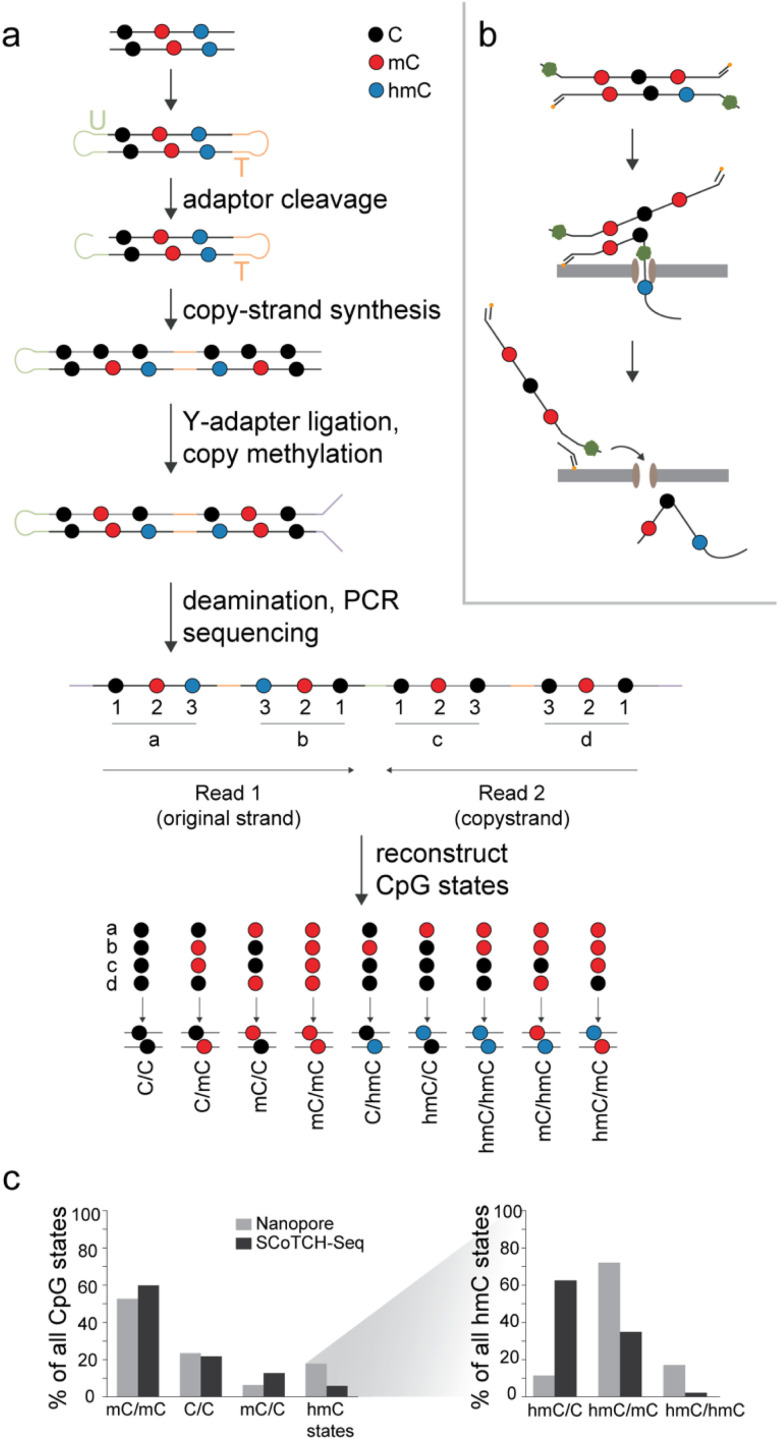
Strategies for simultaneous sequencing of C, mC and hmC enable genomic mapping of individual CpG dyad states. (a) Scheme of the SCoTCH-Seq approach. (b) Scheme of nanopore sequencing with duplex-paired reads. (c) Genomic levels of individual CpG states as reported for nanopore sequencing of mouse cerebellum DNA^28^ and SCoTCH-Seq for mESC DNA.^29^

Recently, the first methods for the selective, simultaneous detection of C, mC and hmC in one experiment run have emerged that provide potential for refined maps with CpG dyad state resolution. These approaches include restriction enzyme-based techniques like DARESOME^42^ and Dyad-seq^27^ that however are limited in coverage and resolution by their dependence on specific restriction sites. Other approaches use complex multistep protocols based on chemical nucleobase conversions to achieve nucleotide resolution with whole genome coverage. For example, the EnIGMA protocol^43^ and its further developed version “SCoTCH-Seq”^29,44^ employ hairpin adapters to store the original DNA sequence and its mC pattern as complement *via* a primer extension and maintenance methylation step, after which hmC and mC are revealed by deamination protocols based either on bisulfite or on multiple enzymatic conversions involving A3A deaminase (Fig. 3a). Another conversion-based approach is SIMPLE-Seq^45^ that employs a K_2_RuO_4_ treatment to oxidize hmC to fC followed by a malononitrile labeling, leading to an adduct that reads as U and is recorded in a complement strand by a primer extension step. Then, the sample is subjected to a TET oxidation step (converting all modified Cs into caC) and subsequent borane reduction, ultimately converting mC in the original template strand to DHU, which results in another C to U transition that can be sequenced. Whether a C to U transition is a result of hmC or mC conversion is decoded by the use of a caC-modified primer in the first primer extension step.^45^ It should be noted that none of the aforementioned methods is designed to sequence the less abundant fC and caC modifications that will read as C or U, as well.

Finally, in addition to restriction- and conversion-based strategies, direct sequencing methods promise particularly simple mapping of modified cytosines.^46^ Here, a very recent study employed nanopore sequencing with duplex-paired reads to map hmC in mouse cerebellum (Fig. 3b) ^28^

So far, only three studies have harnessed one of the aforementioned techniques for establishing genomic maps of individual CpG dyad states. Duplex-paired nanopore sequencing provided highly valuable insights into the locations and overall frequencies of all hmC CpG dyad states in the mouse cerebellum genome that generally exhibits high hmC levels. Symmetric mC/mC was found to be the most abundant state overall, accounting for 53% of all duplex base-calls, whereas symmetric unmodified C/C dyads accounted for 23% (Fig. 3c). Strikingly, hmC-containing dyads occurred predominantly in the form of asymmetric hmC/mCs (72% of all hmC dyads, accounting for 13% of all dyads), which are the first products that TETs generate from their initial mC/mC substrate^28^ (this data roughly aligns with Dyad-Seq data for mESC DNA^27^). In contrast, symmetric hmC/hmC dyads (a direct subsequent TET product) and asymmetric hmC/C dyads (that can theoretically arise from an existing hmC dyad by passive dilution or repair, or from direct off-target oxidation of mC/C dyads by TETs^16^) were found at much lower levels (2–3%, Fig. 3c).

Finally, SCoTCH-Seq has been used for dyad-resolved mapping in mESC genomes, and showed related distributions: mC/mC dyads accounted for 60%, C/C for 22%, mC/C for 13% and all hmC dyads for 6% (Fig. 3c).^29^ Similar to what was observed in the nanopore study, only a very small fraction of hmC dyads were symmetric (2%), whereas 35% existed as hmC/mC and 63% as hmC/C dyads. The orientation of asymmetric dyad modifications was thereby generally random, *i.e.*, did not show strand-bias in respect to gene orientation. An overall conclusion from these studies is that TETs indeed oxidize the two strands of mC/mC dyads independently from each other, corroborating previous *in vitro* studies that indicated a non-processive activity.^22–25^ The studies also refined previous insights into the genomic distribution of hmC. For example, metagene profiles established by SCoTCH-Seq showed an enrichment of hmC/mC and hmC/C in the bodies of actively transcribed genes, and a depletion at transcription start sites, both being significantly more pronounced for hmC/C. Similarly, primed enhancers showed far higher hmC levels than poised or active enhancers that both showed elevated levels only of hmC/C in their flanking regions. This indicates that dyad-resolved maps are extremely helpful for unraveling the function of individual CpG dyad states. With several methods now available at least for parallel C, mC, and hmC sequencing, it is expected that a growing number of dyad-resolved oxi-mC maps will be reported in the near future.

A limitation in mapping dyad modifications is however the inability of current sequencing approaches to extend simultaneous sequencing of oxi-mCs beyond C, mC and hmC. Achieving this maximal chemical resolution will require significant further efforts, but – given the unique properties of fC and caC, and their ability to control interactions with central chromatin proteins – would be highly valuable.^47–54^

Another bottleneck for broader studies is the low levels of many dyad states, resulting in a requirement for costly whole genome deep sequencing. Besides the generally low levels of fC and caC modifications, hmC levels are also low in somatic and particularly cancer tissues.^30,31^ Given the importance of hmC as a cancer biomarker^55,56^ and the still poorly understood functions of fC and caC dyads, future studies would thus greatly benefit from enrichment methods that are applicable to undenatured dsDNA fragments, and offer general selectivity for single cytosine modifications or even for specific dyad modification combinations prior to sequencing.

Current hmC-enrichment strategies typically rely on anti-hmC antibodies^57–60^ that however typically require DNA denaturation and thus destruction of CpG dyad information. Alternatively, T4-β-glucosyltransferase is widely used for enrichment of hmC,^60–62^ whereas engineered MBD proteins have been employed for the enrichment of hmC/mC dyads^63^ and provide a direct and simple access to lower resolution maps.^64^

## 3 The impact of oxidized mC modifications on the physicochemical properties of DNA

**Fig. 4:**
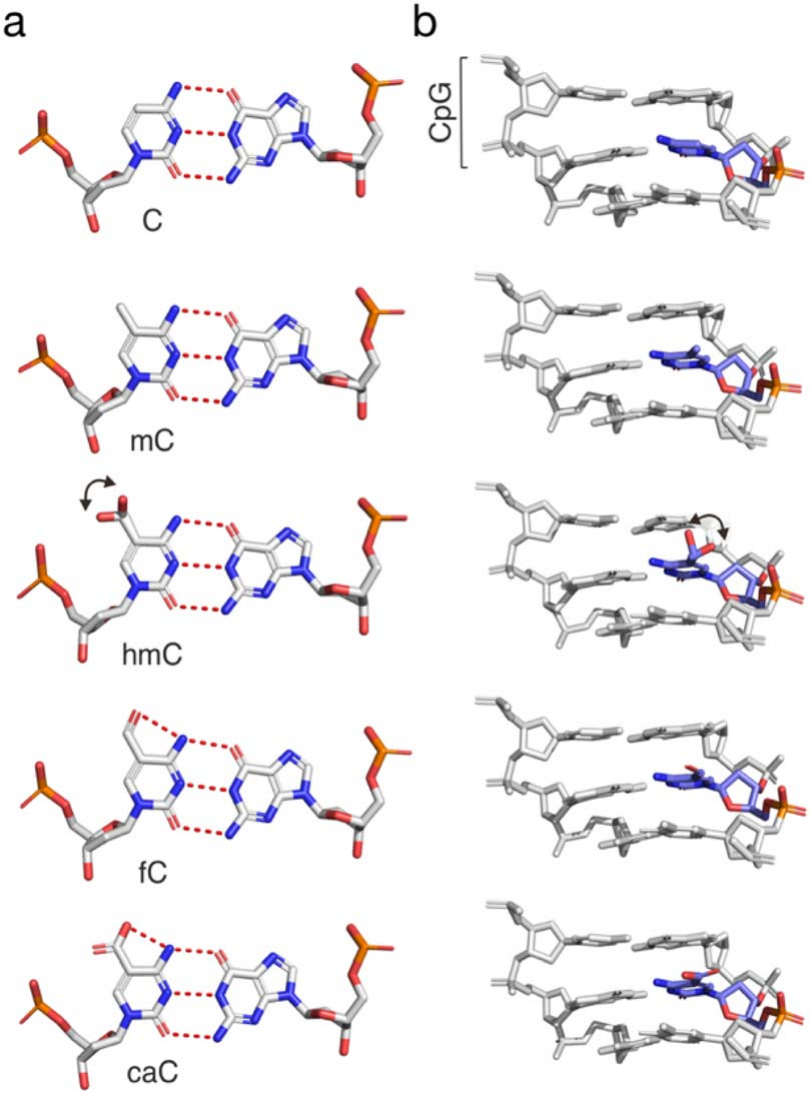
Structures of (a) CG base pairs and (b) trinucleotide duplexes from the Dickerson–Drew dodecamer containing C, mC, hmC, fC, or caC (PDB entries 436D, 4C63, 4I9V, 4QC7, and 4PWM, respectively).^65–67,69^ Hydrogen bonds shown as dotted red lines. Note the conformational freedom of the hmC hydroxyl group indicated by two main orientations in the crystal.

DNA exists in double-stranded form in the mammalian nucleus, and it is complexed with histones and other chromatin proteins. Different CpG dyad states may influence protein interactions in diverse ways, for example, by direct recognition or clash with the substituents themselves, by indirect effects on the electronic properties of the nucleobase that affect hydrogen bonding and stacking, by changes in duplex shape readout *via* altered groove geometries, or by altered duplex flexibility. The 5-substituents differ in hydrogen bonding properties, steric demand and conformational flexibility. The hydroxyl group of hmC is found in crystal structures in a main orientation pointing towards the 3′-nucleobase, and a second conformation undergoing a water-mediated phosphate interaction. In contrast, the fC-formyl and caC-carboxyl groups are fixed in the plane of the cytosine nucleobase, and hydrogen bond to the N4 amino group (Fig. 4a and b).^65–67^ Both fC and caC substantially alter the electronic and chemical properties of the nucleobase, such as charge distribution, polarity, and p*K*_a_ (with caC being negatively charged).^10,68^

Effects of mC itself on local DNA structure have been extensively studied. Among the main findings are an increase of the local curvature of DNA, as well as effects on the groove geometries (slightly widened major *versus* narrower minor groove, respectively). Similarly to mC, hmC has been shown to result in a slight local widening of the major groove^65^ compared to fC- and caC-modified DNA, with the latter exhibiting an enlarged minor groove compared to other modified duplexes in the same study.^70^ However, a thorough solution NMR study with dsDNAs bearing a central CpG in two different sequence contexts afforded structures for C, mC and hmC-modified duplexes that overall showed only modest local effects of the modifications that were smaller than the differences induced by the sequence contexts themselves.^71^ Similar findings were made for fC- and caC-modified DNA.^72,73^

Many aspects of the physicochemical effects of mC and oxi-mCs have been studied as well. Influences on the duplex stability analyzed by thermal melting analyses are highly sequence-dependent. For example, whereas increased TMs observed for a number of mC-modified sequences indicate a generally stabilizing effect,^74–77^ a slight destabilization has been observed for the Dickerson–Drew dodecamer (DDD).^67^ In contrast, hmC has been reported to have either a slightly stabilizing effect (less than mC^71,74,75,77^), a slightly destabilizing effect,^76,78^ or no effect.^67^ Strikingly, either a slightly destabilizing^65^ or no effect^67^ has thereby been reported for the same sequence context (DDD with two different hmC modification settings). Further oxidation of hmC to fC or caC leads to sequence-dependent effects as well. Whereas fC seems to have little impact,^67,77^ caC has been reported to have either a weak^77^ or a strongly stabilizing effect.^67^ In-solution NMR studies with fC- or caC-modified duplexes afforded destabilizing effects in both cases.^72,73^

Effects of the modifications on base stacking have been reported for mC (increased stacking^79–81^), whereas hmC, fC, and caC show similar stacking patterns in crystal structures.^67^

Finally, molecular dynamics simulations and circularization experiments suggest that mC and even more hmC tend to increase DNA stiffness, though again with sequence-dependence.^71^ Single molecule DNA looping studies with varying numbers of modifications were in agreement with a stiffening effect of mC. In contrast, hmC and particularly fC increased the flexibility, whereas caC showed little impact.^82^

Taken together, oxi-mC modifications – while having little effect on the overall conformation of B-DNA in modification patterns studied to date – do partially impact the local duplex structure and can affect the stability and flexibility of dsDNA in a sequence-dependent manner. In light of the observed sequence dependencies and the employment of dsDNA oligonucleotides with single or only a few modification sites in the majority of the aforementioned studies, it is still poorly understood how dense modifications – such as those observed in CpG islands that have high physiological relevance – may lead to more pronounced or even alternative effects. Similarly, the aforementioned studies predominantly focused on DNA containing hemi-modified CpGs. It is therefore not understood, how CpG dyad states may uniquely affect DNA properties, which calls for systematic, comparative studies.

## 4 Proteins reading CpG modification symmetry

Many proteins engage with CpG dyads during their turnover in the cell cycle, and can exhibit selectivity for specific oxi-mC dyad states. Most importantly, this applies to DNMTs,^11,12,83^ TET dioxygenases,^8,18,84^ and TDG^7,10,85^ as factors responsible for the writing and erasing of mC (Fig. 1). In addition, methyl-CpG-binding domain (MBD) proteins, the canonical readers of mC/mC dyads, mediate communication with heterochromatin-associated factors for transcriptional silencing, and show specific dyad state preferences (see below).^3,4^ Whereas MBDs preferentially read symmetric mC/mC CpGs, CXXC and SET- and RING-associated (SRA) domains can recognize the dyad in the non- or hemimethylated state, respectively, and are contained in a variety of chromatin factors. Here, the CXXC of TET3 has been shown to read caC,^51^ whereas the SRA domain of UHRF2 selectively reads hmC.^86^

In addition, fC has been shown to form imine cross-links with lysine/arginine residues of histones and other factors *in vitro*,^49,87^ with potential roles for nucleosome positioning *in vivo*.^48^ As another general factor, RNA polymerase II has been shown to be stalled by fC and caC *in vitro*,^53^ and a specific interaction with caC has been identified in a crystal structure that may account for this effect.^54^ Importantly, beyond such general chromatin factors, oxi-mC dyad states can also modulate the affinity of specific transcription factors (TFs). In the following, we exclusively discuss canonical MBD readers and transcription factors, because of their immediate relevance for the two main regulatory pathways of (oxi)-mC. We will thereby focus on comparative studies involving different oxi-mC dyad states, since only these enable a judgement of actual CpG symmetry functions. Concerning the interactions of other protein factors with oxi-mCs, we refer readers to previous reviews.^88,89^

### 4.1 MBD proteins

The prototypical and best-characterized family of reader proteins for canonical mC/mC CpGs comprises the methyl-CpG-binding domain (MBD) proteins.^90^ The members of the core MBD family in mammals are MeCP2, MBD1, MBD2, MBD3, and MBD4.

Among these, MeCP2, MBD1, and MBD2 exhibit high selectivity for symmetrically methylated CpGs over unmethylated CpGs, and frequently associate with chromatin-modifying complexes such as histone methyltransferases and ATP-dependent remodelers, thereby linking DNA methylation to histone modifications and transcriptional repression.^91^

**Fig. 5:**
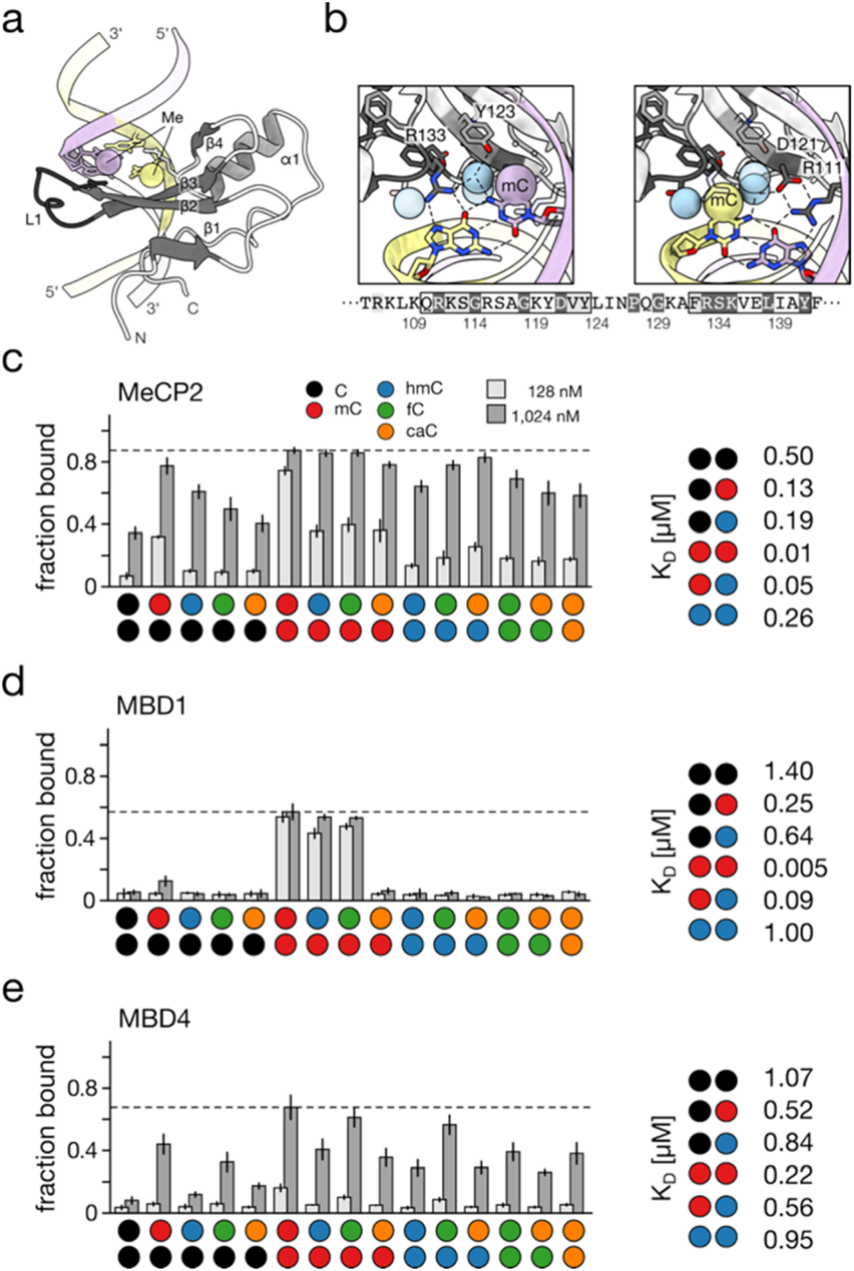
Read-out of CpG dyad states by MBD domains. (a) Overview of crystal structure of the MBD domain of human MECP2 (PDB 3c2i^92^) with methyl groups shown as spheres. (b) Details of G and mC recognition by MECP2 in the two CpG dyad strands. Note the recognition of dyad Gs by conserved Arg residues. Strand coloring as in (a), methyl groups shown as spheres in color according to strand, water shown as blue spheres. Hydrogen bonds shown as dotted black lines. MECP2 protein sequence of shown area indicated below. (c–e) Exemplary binding profiles of MBD domains of human MeCP2, MBD1 and MBD4 to the 15 CpG dyad states (left^93^) and *K*_D_ values for frequent CpG dyad states containing C, mC or hmC (right, MBD4 from mouse).^11^ All data from electromobility shift assays (EMSA). Figure adapted from Buchmuller *et al.*,^63^ in accordance with its creative commons attribution 4.0 international license: http://creativecommons.Org/licenses/by/4.0/.

In contrast, the core family member MBD3 as well as MBD5 and MBD6 diverge in key residues and secondary structure elements, resulting in loss of high-affinity mC recognition.^91^ Structural analyses of MBD–DNA complexes revealed a conserved mode of mCpG recognition. MBD proteins share a small, asymmetric fold that engages the mCpG through two arginine residues hydrogen-bonding to the Hoogsteen face of the CpG guanines (Fig. 5a shows the representative MeCP2-mCpG complex^92,94^). Adjacent residues, including a conserved aspartate and tyrosine, help stabilize these interactions, and form part of hydrophobic pockets that accommodate the two cytosine 5-methyl groups (Fig. 5b). Early reports suggested that hmC is recognized by MBD3 and MeCP2 (ref. 95 and 96). However, subsequent proteomic analyses^97,98^ and comprehensive binding studies failed to confirm these observations. In fact, hmC is bound with markedly reduced affinity by MBD family proteins compared to mC.^11,99,100^ These findings imply that hmC may serve as an intrinsic modulator capable of alleviating mC- and MBD-dependent silencing even without active demethylation. Consistent with this, genome-wide maps correlate hmC with transcriptionally active regions.^62^ Several studies reported broader evaluations of the dyad state preferences of individual MBDs (Fig. 5c–e show exemplary data for three of the five core family MBDs). An overall finding is that all MBDs recognize mC/mC dyads with highest affinity, but with varying degrees of selectivity.^11^ Whereas MBD1, MBD2 and MeCP2 show low nanomolar affinity for mC/mC and comparably high selectivity, MBD4 and particularly MBD3 show lower affinity and selectivity. For the former three MBDs, hmC/mC is the most important off-target (5–18-fold lower affinity than that for mC/mC (Fig. 5c–e^11^). A general tendency among dyads is that mC contributes to high affinity, and that hmC reduces affinity less than non-modified C. fC and caC tend to generally cause similar affinity reductions to hmC, though for MBD1, caC causes significant reductions (Fig. 5d). As visible in Fig. 5c–e, the MBDs overall feature different specificity profiles.^93^ This combined data provides biochemical support for the hypothesis that different oxi-mC dyad states may act as individual regulatory signals, since they may individually modulate a main pathway of mC-dependent chromatin regulation. The advent of dyad-resolved sequencing techniques will help in studying this question in the cellular context.^27–29^

### 4.2 Transcription factors

**Fig. 6:**
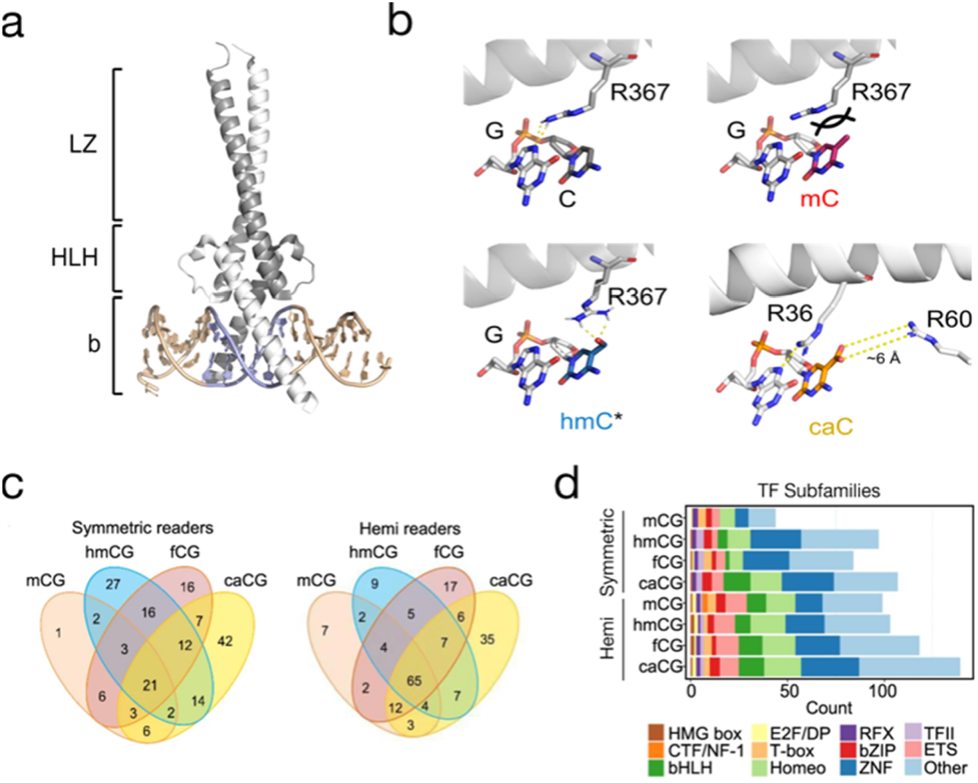
Oxi-mC (anti)reading by transcription factors. (a) Crystal structure of the MYC/MAX heterodimer (PDB 1NKP) bound to E-box DNA. Grey: MAX, white: MYC, blue: E-box. (b) Interaction of MYC R367 in the MYC/MAX dimer with E-box guanine 4 paired with C (PDB 1NKP), models of MYC/MAX bound to mC/mC or hmC/hmC dyads, respectively,^107^ and interaction of MAX R36 and R60 in the MAX_2_ dimer with the caC/caC dyad (PDB 5EYO; *interaction with hmC has not been observed in the E-box sequence). (c) Venn diagrams show readers for mC, hmC, fC, and caC modifications in symmetric and hemi-modified CpGs. (d) The readers of symmetric and hemi-modified CpGs are observed for many TF subfamilies. Figure adapted from Song *et al.*,^108^ in accordance with its creative commons attribution 4.0 international license: http://creativecommons.Org/licenses/by/4.0/.

Many transcription factors recognize target sequences containing a CpG, and they can either be repelled or attracted by mC.^101^ Oxi-mCs equip the dyad with additional information that can be (anti)read by TFs. Examples for TFs that prefer oxi-mCs and for which detailed biochemical and partially structural information is available are SALL1 and 4, which both possess a C_2_H_2_ zinc finger domain with a preference for hmC, and are involved in the recruitment of TET dioxygenases to promote hmC oxidation.^102^ Wilms tumor protein 1 (WT1), another zinc finger TF that is found mutated in nephroblastoms, has been shown to prefer C, mC or caC over hmC or fC.^103^ The presence of hmC also increases the binding of the basic helix–loop–helix (bHLH) TF TCF4 to E-box motif sequences.^104^ Finally, MAX, another bHLH TF and dimerization partner of the master regulator MYC (Fig. 6a), has been shown to bind its E-box target sequence in the presence of a caC or C rather than mC, hmC or fC. Both symmetric and hemi-modified states were characterized, revealing individual affinities for each dyad. A crystal structure revealed a conserved arginine involved in recognition of the caC-paired G residue, and a second arginine in a ∼6 A distance to the caC carboxyl group that may be involved in water-mediated interactions (Fig. 6b).^52^

Besides studies dedicated to specific TFs, pull-down/proteomics screens have been particularly powerful in discovering TFs with general oxi-mC preferences (for overviews of (anti)reader candidates from such studies, see ref. 97, 102, 105, and 106). An overall observation in these experiments has been that fC and caC attract a higher number of readers than hmC. Importantly, the potential of proteomics in this field is still largely untapped, since previous studies employed probes exclusively containing symmetric CpG states. Besides the higher number of theoretically existing asymmetric dyad states (Fig. 2c), symmetric states may also be underrepresented (*e.g.*, in mESC, only ∼2% of hmC is symmetric, whereas 98% resides in hmC/C and hmC/mC dyads; Fig. 3c^27–29^). A recent study reported proteomics screens with DNA promoter probes containing the symmetric and asymmetric CpG states C/C, mC/mC, hmC/hmC, hmC/mC and hmC/C.^107^ Each probe version attracted a different set of readers, and a significant overlap between hmC/C *vs.* C/C and hmC/mC *vs.* mC/mC states hinted at the importance of the canonical C and mC bases for affinity. TFs with verified dyad state preferences included RFX5, MYC and MAX. Interestingly, the two latter proteins showed a preference for hmC in the form of MAX_2_ homo- or MYC/MAX heterodimers, and a possible hmC–Arg interaction was proposed by a model (Fig. 6b). Nevertheless, the specific sequence in which this hmC reading occurs is still unknown (it did not occur in the E-box itself).^107^ Moreover, a previous detailed study covering symmetric and hemi-modified CpG dyad states and the MAX_2_ homodimer found differential read-out of different dyad states. Most importantly, caC was recognized within the E-box sequence with high affinity, and a crystal structure revealed involvement of the structurally conserved arginine (in addition to another arginine, Fig. 6b).^52^

Another caveat of proteomics screens is that pull-down probes typically cover a short, specific DNA sequence (*e.g.*, a promoter fragment), and thus do not contain all possible CpG contexts that may actually be bound by TFs. An improvement in this regard is advanced probe designs with a high density of TF target sequences.^106^ In addition, a complementary, TF-centric approach named digital affinity profiling *via* proximity ligation (DAPPL) has been reported which allows for sampling all possible CpG sequence contexts in a high throughput assay involving >1000 recombinant human TFs.^108^ Here, TF-GST fusion constructs are immobilized on GSH beads, and the GST is tethered to a dsDNA oligonucleotide. Then, a library of dsDNAs containing a central N_8_CGN_8_ sequence is incubated with the pooled TF-GST-bead collection, allowing for a ligation of the two dsDNAs in case of a binding event. Ligation products contain a TF-specific and a CpG dyad state-specific barcode, and can be amplified and pool-sequenced.

With this approach, consensus sequences and CpG state preferences for symmetric and hemi-modified CpGs containing mC and all oxi-mCs have been established for all involved TFs. In general, a higher number of readers was identified for hemi-modified as compared to symmetric CpGs (Fig. 6c), and readers spanned all major TF subfamilies (Fig. 6d). Interestingly, some CpG states could either increase or decrease the affinity of TFs dependent on the sequence context, with examples being PKNOX2, ETS1, or MLX. Taken together, these studies illustrate how specific oxi-mC dyad states can modulate important TF-DNA interactions (including their sequence preferences), which may serve as a basis for regulatory functions *in vivo*. An extension of proteomics screens to additional sequences and of the DAPPL approach to other dyad states would enable a more complete picture of this aspect.

## 5 Conclusions

Cytosine methylation is an essential regulator of mammalian chromatin, with important functions in cell differentiation, development and cancer. Oxi-mCs add another layer of complexity to this regulation which extends beyond their role as active demethylation intermediates. Each oxi-mC decorates the DNA major groove with a sterically unique and polar (in case of fC even electrophilic) functional group that provides unique regulatory potential. The functions of oxi-mCs reside on their influence on DNA's physicochemical properties, their interactions with chromatin proteins, and their genomic locations. A wealth of information is now available about these aspects in general. However, much less is known about the individual properties of different CpG dyad states, owing to a lack of broad, comparative studies. The ability to simultaneously sequence and map C, mC, and hmC CpG states will greatly contribute to a better understanding of their individual functions. In contrast, the impact of different CpG states on the stability, structure, and dynamics of the DNA duplex itself (particularly in the context of physiologically relevant sequences, such as densely modified CpG islands) is still poorly understood, and will require broader studies. First interactomes of individual CpG states are now available from proteomics and *in vitro* selection studies, but these are still far from complete in view of the sampled dyad states and sequence contexts. Most importantly, studying the actual physiological relevance of any of the above findings remains difficult. Whereas dyad-resolved sequencing/mapping methods represent a major step towards meaningful correlation studies, the mixed local occurrence of multiple CpG states and the comparatively poor resolution of complementary mapping methods (*e.g.*, ChIP-Seq) complicate the situation. Moreover, no perturbation methods are available to selectively induce particular dyad states *in vivo*. Given that an increasing number of studies indicate that different oxi-mC CpG dyad states are not functionally equivalent, and that they represent unique regulatory information, broader studies (particularly *in vivo*) are urgently required to fully unravel their individual functions in normal and disease states.

## Author contributions

Z. V. C., L. E. and D. S wrote the manuscript.

## Conflicts of interest

There are no conflicts to declare.

## Data Availability

No primary research results, software or code have been included and no new data were generated or analysed as part of this review.
